# Rethinking the Citric Acid Cycle: Connecting Pyruvate Carboxylase and Citrate Synthase to the Flow of Energy and Material

**DOI:** 10.3390/ijms22020604

**Published:** 2021-01-09

**Authors:** Dirk Roosterman, Graeme Stuart Cottrell

**Affiliations:** 1 Independent Scholar, 48291 Telgte, Germany; 2 School of Pharmacy, University of Reading, Reading RG6 6AP, UK; g.s.cottrell@reading.ac.uk

**Keywords:** Citric Acid Cycle, malic acid, succinic acid, oxidative phosphorylation, exercise, memory formation

## Abstract

In 1937, Sir H. A Krebs first published the Citric Acid Cycle, a unidirectional cycle with carboxylic acids. The original concept of the Citric Acid Cycle from Krebs’ 1953 Nobel Prize lecture illustrates the unidirectional degradation of lactic acid to water, carbon dioxide and hydrogen. Here, we add the heart lactate dehydrogenase•proton-linked monocarboxylate transporter 1 complex, connecting the original Citric Acid Cycle to the flow of energy and material. The heart lactate dehydrogenase•proton-linked monocarboxylate transporter 1 complex catalyses the first reaction of the Citric Acid Cycle, the oxidation of lactate to pyruvate, and thus secures the provision of pyruvic acid. In addition, we modify Krebs’ original concept by feeding the cycle with oxaloacetic acid. Our concept enables the integration of anabolic processes and allows adaption of the organism to recover ATP faster.

## 1. Introduction

“All science is either physics or stamp collecting” [1]. Stamps can be sorted by country and year, or more simply by the value of the stamp. The same is true for enzymes. For example, enzymes of the glycolytic pathway can be aligned by year of discovery or, more classically, they can be didactically aligned based on the gradual degradation of the carbon backbone of glucose. However, we must ask ourselves whether this method of sorting enzymes is correct and how far has this sorting has brought us in understanding Biology. The big difference between Numismatics and Biology is that nature already has sorted enzymes. Nature has sorted glycolytic enzymes strictly in line with the laws of nature and physics. At the time the metabolic enzymes were identified and aligned, some laws of nature, such as the Glansdorff–Prigogine principle, were unknown [2]. Thermodynamically, the body can be considered as a dissipative structure, and the body must compensate for the production of entropy by emitting entropy. Put simply, and in line with the tentative Fourth Law of Thermodynamics, a flow of energy or material is sufficient to form ordered structures [3,4]. It is increasingly clear that enzymes in biological systems exist as ordered complexes and cannot be considered as individual entities catalysing biological processes. Energy entities flow through organisms, sorting enzymes into efficient complexes and organizing cell compartments, metabolons and cell-cell interactions. Glucose metabolism is the primary source of energy and material and is therefore the driving force of this self-organization.

Interestingly, the principle that Biology produces entropy represents the diametrical opposite of the actual common understanding in Biology. This common understanding is deduced from chemistry and experimentally supported by experiments using isolated enzymes. In Chemistry and Biochemistry, concentration gradients are understood to drive reactions. Adding a catalyst or, in the case of biochemistry, an enzyme, to the reaction does not change the equilibration between substrate and product but accelerates the process. Such processes catalyse maximal entropy. Entropic decay is another word for death. In life or Biology, emitting entropy produces concentration gradients. In 1956, R. Crane characterized the Na^+^/glucose symporter [5]. Using biochemical experiments, he showed that the Na^+^/glucose symporter reversibly catalyses maximal entropy or balances a substrate gradient [6]. However, in a biological system, the Na^+^/glucose symporter is connected to the flow of energy and material, and the Na^+^/glucose symporter unidirectionally catalyses the reabsorption of Na^+^ and glucose from pre-urine and participates in the creation of glucose and Na^+^ gradients in organisms. Thus, the Na^+^/glucose symporter acts like Maxwell’s finite being, fighting lifelong entropic decay, and is not Thomson’s demon [7].

Our sorting of metabolic enzymes is in line with the ‘spirit’ of the Glansdorff–Prigogine principle, which is based on the notions of entropy production and energy dissipation. In brief, this means that entropy must be emitted to produce entropy. We have transferred this principle to the molecular and mechanistic levels. However, the translation of Thermodynamics to biological processes requires some changes. Prigogine’s starting point was the Belousov–Zhabotinsky reaction, an isothermal chemical oscillation. Here, one of the reactants is also the product, for example, A + B → 2A [8,9]. Our starting point is the Citric Acid Cycle. The difference here is that oxaloacetic acid is not an oscillating but a cycling product and substrate. The physical quantity for the flow of cycling carboxylic acids was set as a provision, i.e., mol/s. Thus, acting in the ‘spirit’ of the Glansdorff–Prigogine principle means that we do not use the mathematics established in Thermodynamics. Instead, we summarize data and encourage the development of mathematics for biological processes. Biology is all about observation. On the molecular level, the last enzyme of the catabolic pathway, carbonic anhydrase, must transfer the emitted entropy to the first enzyme of the anabolic pathway to produce entropy. The carbonic anhydrase II (CAII)•proton-linked monocarboxylate transporter 1 (MCT1) complex fulfils this function perfectly [10,11,12]. Furthermore, the mechanism of the proton transport chain (PTC) hypothesis also fits well [13]. Here, the active proton (H^+^) of carbonic acid (H_2_CO_3_) is an energy entity. We all know that when acids react with water, the hydration energy is released, producing an exothermic reaction. Thus, entropy doubles. What if the energy of the active H^+^ was saved for a coupled enzymatic step and the acid was channelled (in the absence of water) to the cooperating enzyme? If this were the case, we would achieve a biological process with optimized exergy or minimal entropy. In addition, a water-free transfer of the substrate mathematically would provide an infinite concentration of the substrate and an infinite concentration would drive a coupled enzymatic reaction unidirectionally. In this scenario, it is likely that CAII would position the active proton of H_2_CO_3_ for the transfer to proton-linked MCT1. Thus, CAII would decelerate the Brownian or random motion of H_2_CO_3_ for energy transfer or, in other words, CAII would stop time for the H_2_CO_3_ molecule to increase the possibility of proton (energy) transfer.

In this review, mitochondrial pyruvate carboxylase (PC) and citrate synthase are connected to the ordering flow of energy and material. The connection is accomplished by adding the enzymes that catalyse the membrane transfer of the substrates. The mitochondrial pyruvate carrier (MPC) and heart lactate dehydrogenase (LDH-h)•proton-linked MCT1 complex are both possible candidates to provide pyruvic acid (pyrH) to PC. However, here we discuss LDH-h•proton-linked MCT1 as most likely candidate. It is well understood that LDH-h catalyses oxidation of lactate (lac^−^) to pyruvate (pyr^−^). However, we postulate that LDH-h recovers activity by a NADH-H^+^/NAD^+^ exchange with electron transport chain (ETC), and this exchange acts as a feedback mechanism.

## 2. The Proton Transport Chain Hypothesis

The concept of proton transport chains (PTCs) was deduced from the substrate-channelling hypothesis, which was experimentally demonstrated by Srivastava and Bernhard [14,15,16]. Critics of the substrate-channelling hypothesis have argued that free diffusion minimizes any effects possibly provoked by substrate channelling [17]. We do not agree with this criticism for the following reasons. First of all, the substrate discussed to be transferred between two dehydrogenases is NADH [16]. Instead, we assert that the energy-rich NADH-H^+^ is product/substrate of the dehydrogenases. If free diffusion of the acid NADH-H^+^ into the cytosol occurs, as an acid, NADH-H^+^ will react with water to yield H^+^[H_2_O]_n_. Consequently, free diffusion of NADH-H^+^ entails the change from an active acid to an inactive salt. During reduction processes, it is clear that NADH-H^+^ transfers two H. Therefore, a rationale based on free diffusion would lead to inactivation of the reducing capacity of the co-enzyme NADH-H^+^. Second, the binding affinity of dehydrogenases for the co-enzyme excludes a concept based on free diffusion [18]. The binding of NADH-H^+^ to an enzyme frees the co-enzyme from the hydration layer. It is exactly this water-free binding of substrates to enzymes that allowed us to develop the PTC hypothesis. The term [mol/L] only applies to dissolved substrates. When bound to an enzyme, a substrate is no longer part of the aqueous layer, and both the position and movement of the substrate are defined, not random. This precise positioning stabilizes a specific substrate conformation, one prerequisite for optimal enzymatic activity.

Our PTC hypothesis ensures that a water-free, intra-complex transfer of NADH-H^+^ maintains the activity of the co-enzyme. The PTC hypothesis completely changes the overriding perspective of biological processes based on emitting entropy to a model of producing entropy. In addition, the changes include the mathematical models used to calculate enzyme kinetics. For example, water-free conditions (mathematically) entail an infinite concentration [mol/L] of the substrate and thereby exclude the application of a great number of commonly used mathematical formulae used to calculate enzyme kinetics. An infinite concentration changes enzyme kinetics from concentration dependency to complex kinetics and provision of substrate (mol/s).

In vivo, enzymes such as muscle lactate dehydrogenase (LDH-m) and LDH-h act unidirectionally [19]. However, when they are investigated as isolated enzymes in vitro, they are disconnected from the flow of energy and material and act in a reversible manner. The PTC hypothesis provides a mechanism explaining the observations in vivo that are not reproducible using traditional methods in the laboratory. The PTC hypothesis also integrates the well-known fact that in vivo, enzymes exist as organized complexes. Thus, we assert that the water-free NADH-H^+^ transfer from the proton donor, glyceraldehyde 3-phophate dehydrogenase (GAPDH) to the proton acceptor, LDH-m unidirectionally drives the reduction of pyr^−^ to lac^−^. Thus, in vivo, LDH-m (in complex with GAPDH) only catalyses in the opposite direction the name of the enzyme suggests [20,21]. Citrate synthase is traditionally thought to be an enzyme that catalyses in only one direction. However, a couple of recent studies have reported citrate synthase enzymes capable of catalysing reversibly [22,23]. The enzymes characterized are both from bacteria, namely a sulphur-reducing anaerobic deltaproteobacterium, *Desulfurella acetivorans* [22], and a chemolithotrophic bacteria, *Thermosulfidibacter takaii* ABI70S6^T^ [23]. As bacteria do not have mitochondria, it is extremely difficult to draw comparisons with what happens in a eukaryotic cell. Metabolic enzymes within a eukaryotic cell are compartmentalized, separated by multiple membranes, exposed to different pH levels and are known to exist in enzyme complexes. *T. takaii* ABI70S6^T^ acquires the carbon for metabolic processes from CO_2_ in their environment. Therefore, considering that eukaryotes generate CO_2_ from glucose acquired from their environment, it is not surprising that these bacteria possess a citrate synthase capable of driving a reverse TCA cycle. Interestingly, characterization of the enzymes was performed in vitro using recombinant enzymes. Moreover, anaerobic respiration was performed via fumarate reductase and not via succinate dehydrogenase (SDH)/complex II of the Citric Acid Cycle. Finally, we do not believe that the discovery that bacterial citrate synthase enzymes act reversibly in vitro has any bearing on the eukaryotic Citric Acid Cycle concept. First, the eukaryotic enzyme is present on the human chromosome 12q13.3 and not a product of the mitochondrial genome. Therefore, it has likely diverged from its bacterial ancestor. Second, the condensation reaction catalysed by eukaryotic citrate synthase is practically irreversible, as it has a Δ*G*^0^′ of −7.7 kcal/mol (−32.2 kJ/mol) [24].

Combining enzyme complexes with PTCs creates a new and completely different understanding in Biology. The well-established didactically based sorting of glycolytic enzymes suggests single enzymes perform glycolysis, whereas our concept organizes enzymes into complexes optimizing energy and material transfer. A critical step in applying the tentative Fourth Law of Thermodynamics to biological processes is the identification of the nature of the energy entity, ordering organisms. Glucose metabolism permanently creates the energy entity H^+^. The energy of a H^+^ is high. The absolute hydration free energy of the proton, ΔG_hyd_(H^+^), has been quoted in the literature to be from −252.6 kcal/mol to −262.5 kcal/mol, which corresponds to approximately 35-times the energy released by the hydrolysis of 1 mol of ATP [25]. Thus, H^+^ is always solvated, usually disolvated [26], and ‘free’ protons only exist in a chemical reaction written on a piece of paper. We illustrate this by combining H^+^ with proton carriers, such as H^+^[H_2_O]_n_, NADH-H^+^, lactic acid (lacH), pyrH and H_2_CO_3_.

## 3. Proton Transport Chains in Glycolysis

**Figure 1:**
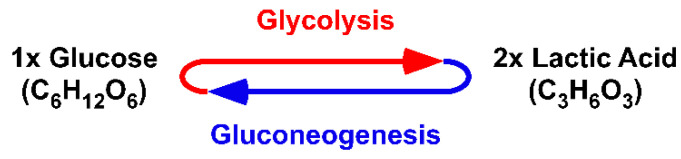
Glucose metabolism as an equilibrium. One molecule of glucose is degraded to two molecules of lactic acid by the process of glycolysis. The reverse synthetic reaction, gluconeogenesis is the conversion of lactic acid to glucose. Meyerhof proposed that the breakdown and synthesis were in equilibrium [28].

Driven by a PTC, the cytosolic GAPDH•LDH-m complex unidirectionally reduces pyr^−^ to lac^−^. Thus, in aerobic or anaerobic conditions, lac^−^ is always formed as intermediate product of glucose breakdown [27]. However, as the original work of O. F. Meyerhof from 1927 showed and as the principle of mass conservation dictates, glucose (C_6_H_12_O_6_) is metabolized to two molecules of lacH (C_3_H_6_O_3_) (Figure 1) [28].

Consequently, one of the well-known chemical reactions of glycolysis is incorrect. Somewhere en route from glucose to lacH, two protons are lost. In our PTC hypothesis, we suggest that the intermediate product of the phosphoglycerate kinase (PGK)-catalysed reaction is an acid carrying the defalcated H^+^ [13]. In science, these two H^+^ mark the line between Chemistry and Alchemy. Atoms cannot just appear or disappear, otherwise we would study alchemy. The defalcated H^+^ changes everything. PGK is located at both the plasma membrane and in the cytosol depending on ATP and NADH levels [29]. At the plasma membrane, we postulate that PGK and the proton-linked MCT4 form a complex [13]. The permanent catalytic activity of PGK permanently provides the intermediate glucose breakdown product, 3-phosphoglyceric acid. The permanent provision of 3-phosphoglyceric acid in the immediate proximity of proton-linked MCT4 fulfils the observed biological function of proton-linked MCT4: The export of monocarboxylic acids [30,31]. Thus, the PGK•proton-linked MCT4 complex exports monocarboxylic acids depending on the glycolysis rate. In contrast, the CAII•proton-linked MCT1 complex imports monocarboxylic acids depending on the rate of oxidative phosphorylation or H_2_CO_3_ emission. Our sorting of enzymes is based on the subcellular location and a coupling partner. Thus, the energy generated by glucose breakdown or emitting H_2_CO_3_ is channelled to initiate membrane transfer of monocarboxylic acids. Our sorting of metabolic enzymes not only changes the well-known flowchart of glycolytic enzymes, but also opens up avenues to understand the mechanisms regulating complex biological processes [32].

## 4. Proton Transport Chains and Citric Acid Cycles

The rationale that NADH does not have the ability to transfer two H molecules questions the well-established net chemical formula of the mitochondrial pyruvate dehydrogenase complex (PDHc) found in standard textbooks. Some textbooks present this stoichiometrically correct chemical formula for the PDHc-catalysed reaction (see 1):

CH_3_C(=O)C(=O)O^−^ + HSCoA + NAD^+^ → CH_3_C(=O)SCoA + NADH + CO_2_ (1)

However, in some others, one of the lost protons magically reappears in the form of NADH-H^+^ (see 2).

CH_3_C(=O)C(=O)O^−^ + HSCoA + NAD^+^ → CH_3_C(=O)SCoA + NADH-H^+^ + CO_2_ (2)

The reason behind its appearance is logical when the next chapter of the textbook discusses the oxidation of NADH-H^+^ during oxidative phosphorylation. As the ETC depends on NADH-H^+^, we assert that PDHc depends on pyrH. Our reasoning is as follows. The first step of the PDHc-catalysed reaction is a nucleophilic substitution. Basic chemistry knowledge excludes the possibility of a nucleophilic substitution on a negatively charged thiaminpyrophosphate (TPP) targeting a negatively charged anion, pyr^−^ [13].

A scientifically based chemical formula of the PDHc-catalysed process sets charge-neutral pyrH as the target of TPP. Indeed, Lester Reed published this chemical formula from 1960–1990. PDHc belongs to the family of α-ketoacid dehydrogenase [33,34]. Only using pyrH as substrate can PDHc activity lead to the formation of biologically active NADH-H^+^ (see 3). The addition of a H^+^ to the PDHc catalysed process means that TPP can substitute the partially positively charged α-carbon of pyrH and biologically active NADH-H^+^ is formed. Thus, the equation for PDHc becomes:

CH_3_C(=O)C(=O)O**H** + HSCoA + NAD^+^ → CH_3_C(=O)SCoA + NADH-H^+^ + CO_2_ (3)

PDHc is located at the inner mitochondrial membrane in the mitochondrial matrix. As such, the substrate of PDHc must be provided through the mitochondrial membrane. Charge-neutral pyrH does not induce an electric potency blocking membrane transfer, strongly suggesting that pyrH, not pyr^−^, is transferred through the membrane. An alternative hypothesis sometimes suggested in textbooks is that H^+^ is provided by the mitochondrial lumen and the anion pyr^−^ passes through the membrane. Quite interestingly, two mitochondrial pyrH transport enzymes have been characterized. This immediately suggests to us the existence of two distinct Citric Acid Cycles. Recently, the MPC was cloned and characterized [35,36]. MPC feeds the well-known pyr^−^-Citric Acid Cycle with the substrate pyrH. Quite unrecognized, but characterized years before MPC was cloned, is the lac^−^-Citric Acid Cycle. The mitochondrial-located LDH-h•proton-linked MCT1 complex oxidizes lac^−^ to pyr^−^, which is transferred as pyrH to PDHc. Investigation of the kinetics of proton-linked MCTs has clearly shown a three-step reaction. First, the energy (H^+^) is transferred to proton-linked MCT. Second, carboxylates bind. Finally, the acid is charge-neutral transferred through the membrane. Characterization of the family of proton-linked MCTs clearly demonstrates that the carboxylic acid is charge-neutral transferred through the inner mitochondrial membrane to PDHc [37,38,39]. In addition, this process provides one molecule of NADH-H^+^ [40].

In line with the concept that emitting entropy is needed to produce entropy, we postulate that the emitting product of the PDHc-catalysed reaction, CO_2_, is hydrated to H_2_CO_3_. The nascent H_2_CO_3_ is then channelled through the mitochondrial membrane and provides an active H^+^, driving the import of the substrate of PDHc [38]. Similarly, oxidation of lac^−^ to pyr^−^ provides an active proton in the form of NADH-H^+^. It is rational to suggest an exchange of the substrate of the ETC, NADH-H^+^, with the end product of the ETC, NAD^+^, which recovers LDH-h activity [13]. The suggested NAD^+^/NADH-H^+^ exchange is understood as a regulatory feedback mechanism. An imbalance in the NAD^+^/NADH ratio is sometimes associated with diseases. The provision of energy, NADH-H^+^, is the initiating step of energy-driven enzyme complexes. Thus, an imbalance indicates the shortage of the substrate of the coupled enzymatic reaction or the accelerated flow of the energy-providing pathways.

## 5. Connecting Mitochondrial Pyruvate Carboxylase and Citrate Synthase to the Flow of Energy and Material

Following our brief summary of the PTCs and the importance of sorting of enzymes in line with the flow of energy, we now explain how we connect PC and citrate synthase to the flow of energy and material. If enzymes are sorted in the ‘spirit’ of the Glansdorff–Prigogine principle, then we obtain enzyme pairs. Each pair is designed such that one enzyme emits entropy and the coupled enzyme produces entropy. We have already mentioned a specific example, the CAII•proton-linked MCT1 complex. This complex couples the permanently emitted H_2_CO_3_ to the creation of a cytosolic monocarboxylic acid gradient. The huge difference in the mechanism, or the underlying principle of the well-established sorting and our sorting of metabolic enzymes, is that the well-established concept understands the created monocarboxylic acid gradient as already sufficient to drive gluconeogenesis and fatty acid synthesis. In contrast, our sorting predicts that every reaction of a metabolic pathway producing entropy must be linked with an entropy emitting reaction.

**Figure 2:**
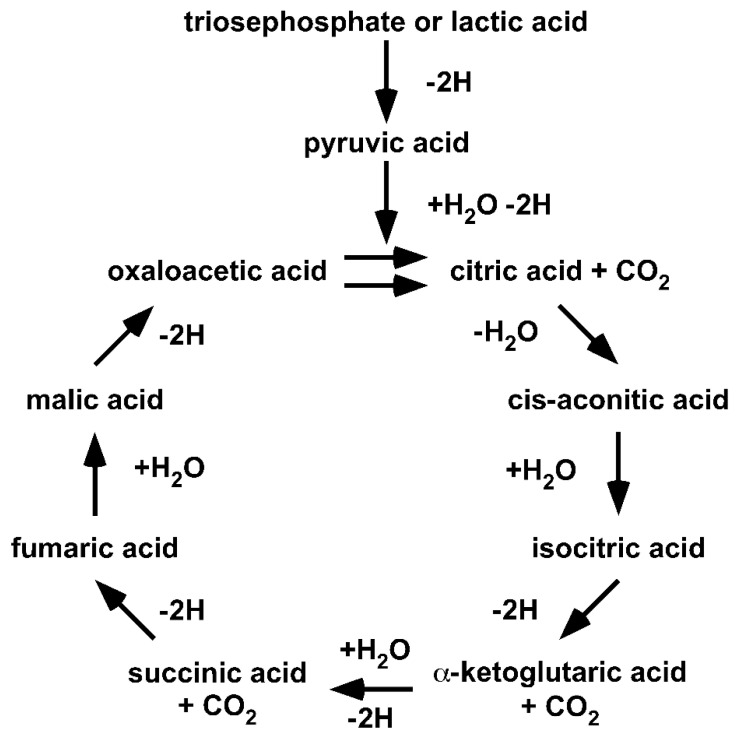
Krebs’ Original Citric Acid Cycle. This illustration is taken from H. A. Krebs’ Nobel Prize lecture in 1953 and first published 1937 [41,42,43]. H. A. Krebs received the Nobel Prize for his discovery of the Citric Acid Cycle. ©The Nobel Foundation.

Since Krebs’ formulation of the Citric Acid Cycle, a large number of diverse changes have been introduced into the cycle [41,42]. A Google search for “Krebs cycle” provides more than one million hits and many subtly different cycles. The diversity in individual understandings of Krebs’ Citric Acid Cycle makes it necessary to return to the origin. Thus, we reset our understanding to the year 1953 (Figure 2).

Returning to the origin demonstrates that (I) the cycle is fed by lacH, not pyr^−^; (II) the cycle handles carboxylic acids, not carboxylates; (III) the cycle only proceeds in a unidirectional clockwise direction; and (IV) the cycle illustrates the degradation of lacH to 2H, CO_2_ and H_2_O. It should be obvious that the original Citric Acid Cycle is a purely degradative cycle. Krebs illustrated that oxaloacetic acid and, in fact, all acids of the Citric Acid Cycle are cycled within the cycle. Thus, all of the acids are intermediates and none can be given to anabolic pathways. The only way an anabolic pathway can be integrated is if the cycle is fed with oxaloacetic acid.

Within the one million hits of the Google search for “Krebs cycle,” cycles showing enzymes acting reversibly instead of unidirectionally, cycles showing carboxylates instead of carboxylic acids and cycles fed with pyr^−^ instead of lacH are included. Furthermore, anabolic pathways have become ‘attached’ to a purely catabolic cycle. It is fair to say that over the last 80 years, the Krebs’ Citric Acid Cycle has become a victim of Babylonian language confusion or the freedom of science.

**Figure 3:**
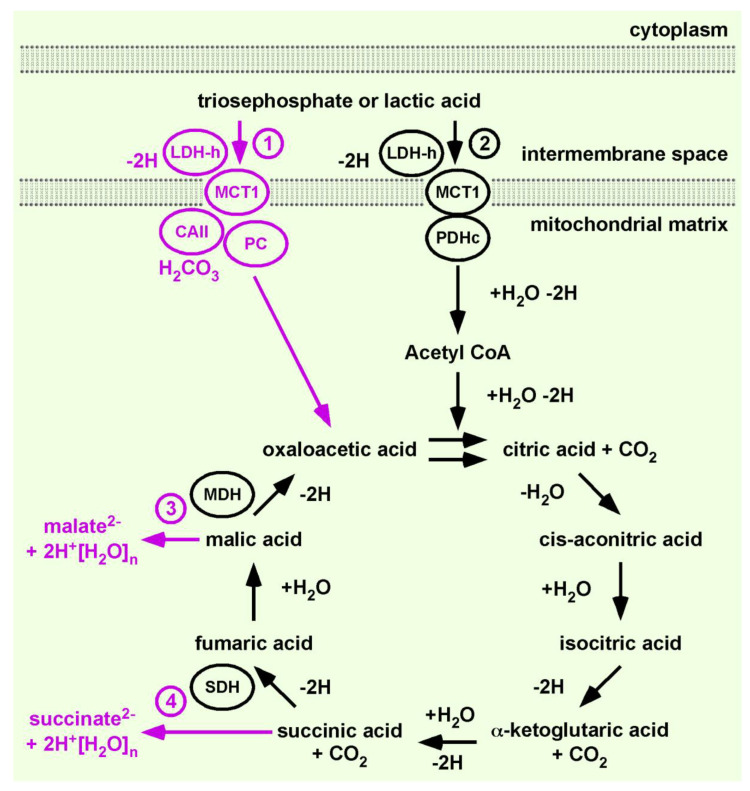
Citric Acid Cycle 1.1. ① The heart lactate dehydrogenase (LDH-h)•proton-linked monocarboxylate transporter 1 (MCT1) complex, which catalyses the first reaction of oxaloacetic acid synthesis and the Citric Acid Cycle 1.1, is added to the cycle (magenta). Carbonic anhydrase II (CAII) provides the flow of carbonic acid (H_2_CO_3_) necessary for the proton-linked MCT1 catalysed membrane transfer of pyruvic acid. Pyruvate carboxylase (PC) then converts pyruvic acid to oxaloacetic acid. ② Concomitantly, the LDH-h•proton-linked MCT1 complex transfers pyruvic acid to the pyruvate dehydrogenase complex (PDHc), which provides Acetyl CoA to the Citric Acid Cycle 1.1. All acids cycle within the cycle. ③ A relative inactivity of malate dehydrogenase (MDH) causes a ‘metabolic traffic jam,’ and excess malic acid shunted to a mitochondrial pool of carboxylates as malate^2−^. ④ A relative inactivity of succinate dehydrogenase (SDH) regulates the release of succinic acid into the pool of carboxylates as succinate^2−^. Note: This illustration contains an extract from the image shown in H. A. Krebs’ Nobel Prize lecture in 1953 and first published 1937 [41,42,43]. ©The Nobel Foundation.

In order to integrate gluconeogenesis or anabolic pathways into the Citric Acid Cycle, a permanent provision of oxaloacetic acid is obligatory. In order to satisfy this need, we added PC to the Citric Acid Cycle. To differentiate between our continuation of the work of H. A. Krebs from the cycles within the ‘one million’ hits and to indicate that PC expands the function of the original concept significantly, we tentatively named our evolving model the Citric Acid Cycle 1.1 (Figure 3).

To integrate PC, we applied the exact same rationale that allowed the connection of PDHc to the flow of energy and material. The substrate of PC is also pyrH, provided by the LDH-h•proton-linked MCT1 complex, and LDH-h recovers activity via a NADH-H^+^/NAD^+^ exchange with the ETC. This NADH-H^+^/NAD^+^ exchange is a feedback mechanism likely regulating oxaloacetic acid synthesis. PC produces entropy by catalysing the ATP-dependent substitution of H_2_CO_3_ into pyrH to produce oxaloacetic acid.

Given that the most favoured assumption is that the anion, pyr^−^, is substrate of PC, we are aware that the first issue our sorting encounters is claiming that pyrH and H_2_CO_3_ are substates and that oxaloacetic acid is a product of PC. However, the underlying mechanism of PC catalysis is the stabilization of the enol form of pyr^−^. Our understanding of keto-enol tautomerism is that the α-keto group must draw electrons in order to change the molecule into the enol form and that the anion pyr^−^ has excess electrons. The excess electrons exclude the formation of an enol, which can be enzymatically stabilized. Furthermore, as substrate of PC is provided through the mitochondrial membrane, the LDH-h•proton-linked MCT1 complex is a very good candidate to fulfil this job. Thus, we predict that the true substrates of PC are pyrH and H_2_CO_3_ and that the product is oxaloacetic acid.

PC delivers oxaloacetic acid to the Citric Acid Cycle 1.1. We already have discussed that LDH-h•proton-linked MCT1 preferentially feeds the catabolic lac^−^-Citric Acid Cycle. Thus, LDH-h•proton-linked MCT1 provides pyrH and NADH-H^+^, suggested for PDHc and ETC [13]. Now, we add a second LDH-h•proton-linked MCT1 to feed PC and ETC. This progress allows the synthesis of citric acid, or construction of the Citric Acid Cycle. The net chemical formula catalysed by citrate synthase is:

Oxaloacetic acid + Acetyl-SCoA + H_2_O → Citric acid + HS-CoA

H. A. Krebs illustrated the unidirectional degradation of lacH. During this process, NADH-H^+^ and FAD-H_2_ are produced, consumed and recovered by oxidative phosphorylation.

The dominating concept of enzyme kinetics has created the term anti-clockwise or reverse Krebs cycle. The anticlockwise Citric Acid Cycle is a rational product of the well-established concept of enzyme kinetic, suggesting a short cut to malic acid synthesis. An increasing concentration of oxaloacetic acid is considered to change the catalytic direction of malate dehydrogenase (MDH).

It is unrecognized that the nature of this cycle is that product of MDH, oxaloacetic acid, is also a substrate of the cycle. In other words, the well-established understanding that enzymes act reversibly depending on a change in the concentration equilibrium between product and substrate encounters a dilemma. Both the substrate and product of the cycle are similarly changed by the addition of oxaloacetic acid. There are two ways out of this dilemma: by understanding the work of H. A. Krebs, which shows that malic acid is unidirectionally catalysed to oxaloacetic acid, or by trusting what generations of scientists have learned. The latter will surely be verified by an ex vivo experiment showing that MDH reversibly catalyses an equilibrium between malate^2−^ and oxaloacetate^2−^. However, this would then lead to the rational assumption that increasing the concentration of oxaloacetate^2−^ must change the equilibrium of the MDH-catalysed reaction in the direction of malate^2−^.

However, we chose the first option for a number of compelling reasons that we have already discussed. A proton is an energy entity. Substrate channelling in the absence of water ensures the active proton of acids remained undissociated, meaning carboxylic acids and not carboxylates are the substrates. The water-free transfer of acids provides a mathematically infinite concentration, driving a unidirectional reaction. Water-free transfer also ensures that carboxylic acids are charge-neutral transferred through the inner mitochondrial membrane and that the alpha-keto group of pyrH and α-ketoglutaric acid is target of nucleophile substitution. Enzyme complexes are predicted by the Fourth Law of Thermodynamics and the PTC hypothesis [2,13]. The theoretical background for the original Citric Acid Cycle is achieved by transferring this Law of Nature and the PTC hypothesis to a biological process. H. A. Krebs illustrated the Citric Acid Cycle as a unidirectional flow of material driven by the burning of lactic acid to water, carbon dioxide and hydrogen (Figure 2). This is not a matter for discussion. H. A. Krebs was aware that acids dissociate in water and that enzymes catalyse in both directions. Nevertheless, he was honoured with the Noble Prize for his discovery of the unidirectional Citric Acid Cycle. Yet, over time, other scientists began to change the ‘Krebs cycle’ into a carboxylate cycle, misquoting the scientific work of H. A. Krebs [41,42,43].

In this manuscript, we apply the underlying mathematics of enzyme complex kinetics. The mathematics was deduced from a Law of Nature formulated decades after H. A. Krebs formulated the Citric Acid Cycle. In our discussion, the mechanism of malic acid synthesis is introduced and we discuss experimental data and theoretical considerations designed on basis of the original concept and supporting the original concept, respectively.

## 6. Discussion

Our concept of sorting stamps/metabolic enzymes is by no means complete and is still under development. In our recent reviews, we have presented arguments and postulated that the ‘TCA cycle’ must be split into preferentially anabolic pyr^−^-TCA and catabolic lac^−^-TCA/ETC cycles [13,32]. We used the vocable ‘TCA cycle’ automatically. This vocable was learned at school, repeated during study and was used without checking the original source. Working on this review has shown that the postulated lac^−^-TCA/ETC cycle [13] exactly represents the concept that H. A. Krebs presented at his Noble Prize lecture [43]. We understand our integration of PC to the purely degradative cycle as a significant step in understanding and function, as well as in continuing of the work of H. A. Krebs, and suggest the name Citric Acid Cycle 1.1.

We encountered many problems and barriers by resetting the understanding of glycolysis and gluconeogenesis to the year 1927 and following the two defalcated protons [13]. Our hypothesis cannot be followed on the basis of the common understanding of glucose metabolism. However, we found resetting the understanding of the Citric Acid Cycle to 1953 and following two dozen defalcated H^+^ to be much more challenging.

We first attempted to link our hypothesis to actual scientific knowledge by referring to protein crystallization data. A search of the literature unearthed a number of possible substrate combinations for PC. Starting with the characterization of PC by Utter and Keech in 1960, the substrates were suggested to be pyr^−^ and CO_2_. In 1962, Walker suggested pyrH and CO_2_, and it is highly likely that all substrate permutations could found in the pursued research. However, only one combination of substrates is correct [44,45]. We assumed that the correct combination is the only one which can fit into the active site of PC. Actual crystallization data for PC revealed that a protonated arginine in the active site of PC stabilizes the substrate bicarbonate (HCO_3_^−^), which is substituted into the enol form of pyr^−^. Thus, based on actual crystallography data, pyr^−^ and HCO_3_^−^ are the substrates and oxaloacetate^2−^ is the product [46,47]. This data fits perfectly into the actual favoured substrate permutation. We found this extremely interesting, as we would have assumed that H^+^[H_2_O]_n_ is sterically prevented from entering this specific position to transfer H^+^. Although we are not experts in protein crystallography, is it possible that the presented protonation of the arginine residue is an artefact of the purification process demonstrating that H^+^[H_2_O]_n_ can reach the active site to transfer H^+^?

The current understanding of the structure, mechanism and regulation of PC differs greatly from the understanding of 30 years ago. The PC crystallization data of Karpusas and coworkers, published in 1990, revealed a structure resembling a proposed transition state of the condensation reaction. “This structure suggested that the condensation reaction proceeds through a neutral enol (pyrH) rather than an enolate (pyr^−^) intermediate.” Additionally, the reaction catalysed by citrate synthase was discussed to proceed via a Claisen condensation of oxaloacetic acid and acety-SCoA, forming the product citric acid [48]. The Citric Acid Cycle complex is further supported by observations of citrate synthase•PDH and citrate synthase•PC•MDH complexes [49,50]. Interestingly, in the study by Fahien et al., the authors did not find evidence that citrate synthase associated with pyruvate carboxylase. These real but seemingly forgotten data support the Citric Acid Cycle 1.1.

Next, we turn our attention to the well-established assumption that the product of citrate synthase is citrate, presumably leading to the renaming of the Citric Acid Cycle to the ‘citrate cycle.’ Previously, we claimed that the product of PC is oxaloacetic acid. However, let us assume the scenario that the PC•citrate synthase complex releases citric acid into the environment. Citric acid, by reacting with water, undergoes entropic decay into citrate^3−^ and three H^+^[H_2_O]_n_. However, our concept is based on the water-free transfer, not entropic decay, and we support the notion that the substrate and product of citrate synthase are oxaloacetic acid and citric acid, respectively [48]. Additionally, it is rational that the neutral pH of the mitochondrial matrix can only be maintained by water-free channelling of the acids within enzyme complex.

Sorting the enzymes of the Citric Acid Cycle into one complex and applying the PTC hypothesis is not just our way continuing the work of H. A. Krebs on the Citric Acid Cycle, but it also enables the enzymes of the Citric Acid Cycle to catalyse. Interestingly, α-ketoglutarate dehydrogenase together with PDH belong to the family of α-ketoacid dehydrogenases. Thus, if citrate synthase released nascent citric acid into the mitochondrial matrix, the cycle encounters the problem that the substrate of α-ketoglutarate dehydrogenase has to be α-ketoglutaric acid (see above discussion on nucleophile substitution).

S. Nath illustrated the pH dependency for the equilibration between succinic acid, succinate^−^ (mono-anion) and succinate^2−^ (di-anion) [51]. At pH 7, there is an equilibration between succinate^−^ and succinate^2−^. There will be a similar equilibrium between α-ketoglutaric acid/α-ketoglutarate. Thus, transferring this knowledge to α-ketoglutaric acid reveals that at the neutral pH of the mitochondrial matrix, the substrate of α-ketoglutarate dehydrogenase, the acid form, does not exist.

We simply present the curiosity of ancient black-and-white chemical formulae for the following reason. The mitochondrial matrix is structured in cristae or loops. The Citric Acid Cycle 1.1 is one building block for the construction or formation of the mitochondrial structure. Adding proton-linked MCT1 as the first enzyme of the Citric Acid Cycle 1.1 sets the cornerstone. The location and catalytic direction of all enzymes of Citric Acid Cycle 1.1, as well as enzymes of pathways directly (water-free) linked to the cycle, are defined by the setting of the cornerstone. Thus, our stamp collection album is a three-dimensional building, like nature. Buildings have a construction plan for the architects and a glossy photomontage for the buyer. We work solely on the construction plan. An illustration of the three-dimensional structure of the inner-mitochondrial membrane is difficult to convey by aligning the enzyme complexes in a one-dimensional drawing.

Of course, there are tools and databases supporting scientists to prepare illustrations and analyse data. The Kyoto Encyclopaedia of Genes and Genomes (KEGG) database is commonly used to provide a colourful illustration of the enzyme complexes of oxidative phosphorylation [52]. Wrapping this template around the Citric Acid Cycle 1.1, one loop of the inner mitochondrial matrix is formed by itself. Before doing so, it must be mentioned that prokaryotes do not have mitochondria, and fumarate reductase is not a synonym of SDH.

If we then start with our cornerstone, proton-linked MCT1, then NADH dehydrogenase (*Thermus thermophilus*) must be in the immediate proximity of the cornerstone to allow water-free transfer of NADH-H^+^. Next, complex II (fumarate reductase, *Escherichia coli*), a known as an enzyme of an anaerobic Citric Acid Cycle, must be placed next to NADH dehydrogenase. The PTC hypothesis postulates that the co-enzyme NADH-H^+^ does not feed a pool of NADH, as suggested by KEGG. Instead, every generated NADH-H^+^ must be linked to a specific proton donor enzyme complex. Considering that the flow of energy and material forms the mitochondrial structure, an interruption of the flow, hypoxia, must affect the structure stabilized by energy flow [53]. It is obvious that using a KEGG template as basis for discussing the dynamics of inner mitochondrial membrane is not within the scope of this review. Together, this indicates that an extensive literature search is necessary before an illustration can be drawn, and that this illustration will change according to the flow of energy.

Just the suggestion of creating such an illustration would undoubtedly draw us into the middle of the ‘OX PHOS war zone’ [54]. However, our sword and shield in these wars is stoichiometry and the reasonable doubt that ATP synthase is driven by a pH gradient delivering blank protons.

During our research and literature searches, we discovered the work of S. Nath, which clearly demonstrates that F_0_F_1_-ATP synthase does not solely transfer protons. Instead, the substrates of F_0_F_1_-ATP synthase are dicarboxylates^−^. Nath’s two-ion theory of energy coupling challenges the well-established concept of chemiosmotic-driven ATP synthase formulated by P. Mitchell [55,56,57]. Interestingly, malate^−^ is the preferred substrate of ATP synthase during photophosphorylation in chloroplast thylakoids, whereas during oxidative phosphorylation in mitochondria, succinate^−^ is the preferred substrate of F_0_F_1_-ATP synthase.

The Citric Acid Cycle 1.1 opens a pathway to explain how repeated or persistent glycogenolysis in muscle cells or astrocytes increase the availability of the substrates of ATP synthase.

Glycogenolysis greatly increases lacH concentration and, thereby, the cycling (mol/s) of acids within the Citric Acid Cycle [58]. The provision of oxygen (breathing) does not increase at a similar rate. Yet, even 20 min after exercise, the NADH/NAD^+^ ratio greatly increases, indicating an excess of reductant [58]. It is worth mentioning that glycogenolysis is better understood as the overproduction of the reductant rather than the diminished provision of the oxidant. Even if the primary effect of increased cycling of acids is similar to hypoxia, the recovery rate of NADH-H^+^ and FAD-H_2_ is diminished. Thus, an overproduction of the starting fuel (lacH) for Citric Acid Cycle 1.1 results in the longevity of the reduced forms of the co-enzymes and inactivity of MDH and SDH. The substrates of MDH and SDH are malic acid and succinic acid, respectively. However, neither substrate can accumulate within the enzyme complexes. The term ‘metabolic traffic jam’ describes this situation well. The cause of this metabolic traffic jam is an imbalance between the flow (mol/s) of energy and material (driven by the catabolism of lacH) and the flow (mol/s) of energy and material driven by ETC. As consequence of this imbalance, malic acid and succinic acid are ‘pushed’ into the mitochondrial matrix to feed the pool of carboxylates^2−^ [59,60]. It is worth mentioning that we actually understand the Citric Acid Cycle 1.1 to synthesize only malic acid and succinic acid. The other acids are understood to be cycling intermediates.

The presented concept of the Citric Acid Cycle 1.1 introduces, for the first time, a ‘metabolic traffic jam’ as a redox mechanism. It also highlights that, as well as a source of acetyl-SCoA, the cycle also needs an additional source of carboxylic acid (oxaloacetic acid) that is obligatory for the synthesis of carboxylic acids. In turn, the synthesized carboxylic acids act as sources of carboxylic acids for other cycles and pathways, indicating the dynamics of biological processes. Krebs’ Citric Acid Cycle and the Cori cycle (the rebuilding of glucose and glycogen in the liver from muscle lactic acid) are considered as well-established concepts. Investigations of the Cori cycle by chasing radioactive-labelled carboxylic acids in isolated liver or in animal model have demonstrated a wide distribution of the radioactive label, indicating a variety of interchanging reactions [61,62,63]. Thus, neither the Cori cycle nor the Citric Acid Cycle 1.1 represent a linear process than can easily be determined by chasing radioactive labels. The increase of malate in muscle and liver during exercise was the initiating data for the formulation of the ‘metabolic traffic jam’ [60,64]. Experimental data can (I) be collected like stamps, (II) be sorted into site reactions and gluconeogenesis, (III) used to change the initial concept or (IV) functionally linked by physics. Point III is a pitfall. Using and determining carboxylates, such as pyr^−^, malate and citrate, has changed the original Citric Acid Cycle into a plethora of citrate cycles. The data may be compelling, but a Krebs´ citrate cycle violates the Laws of Nature, copyright law, mathematics and basic chemical understanding.

The ‘metabolic traffic jam’ fills the mitochondrial pool of dicarboxylates and allows cells to react faster to environmental stressors. Learning and exercising are the two most famous environmental stressors. Interestingly, both learning and exercising are linked to glycogenolysis in astrocytes and muscle cells, respectively [32]. Glycogen breakdown provides lacH, which is not only ‘food for hungry neurons and muscle’ but, like all glucose metabolites, a primary signalling molecule triggering ATP-, pH- and redox-sensitive signalling pathways [32,65]. Organisms adapt to and memorise environmental stressors. For example, in the spirit of D. O. Hebb, a persistent or repetitive breakdown of glycogen induces lasting cellular changes, including growth processes and metabolic changes [66].

**Figure 4:**
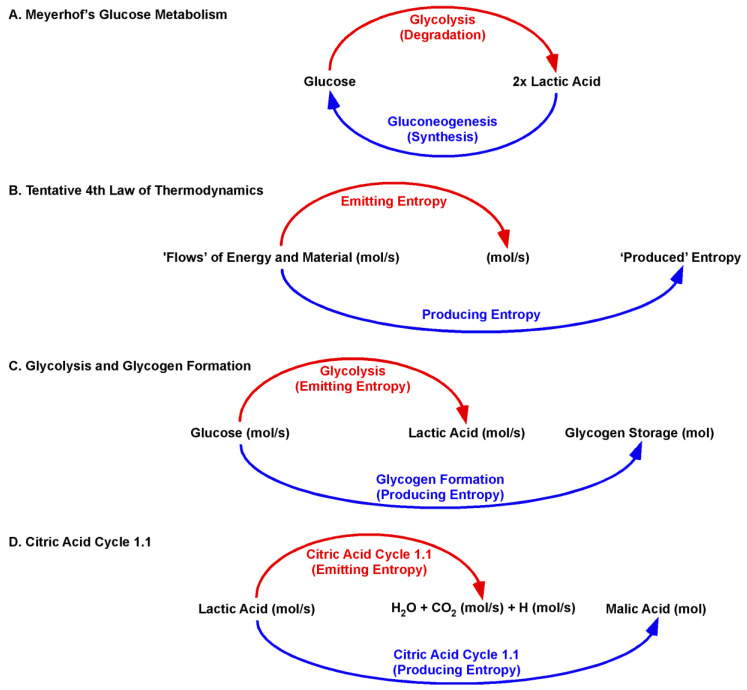
From Chemistry to Biology in metabolism. (**A**) Glucose metabolism is illustrated as a chemical reaction. Meyerhof’s interpretation of the metabolism of glycolysis is based on strict stoichiometry. One molecule of glucose is converted to two molecules of lactic acid. The degradation reaction (glycolysis) and the reverse synthetic reaction (gluconeogenesis) are in equilibrium. (**B**) The tentative Fourth Law of Thermodynamics asserts that a flow of energy and material (emitting entropy) is sufficient to produce entropy. (**C**) By transferring the tentative Fourth Law of Thermodynamics (a law of nature) to Biology, glycolysis (emitting entropy) is sufficient to produce entropy (glycogen). The different physical quantities are key here: By setting glucose transporters as the first enzyme and proton-linked MCT4 as the final enzyme, glycolysis is a flow of energy and material (mol/s), whereas glycogen (mol) is a product of this flow. (**D**) The Citric Acid Cycle 1.1 makes the dynamics clear. If lactic acid is not “burned,” the import of lactic acid into the cycle is blocked.

The Citric Acid Cycle 1.1 replaces the well-established biochemical concepts of metabolism with a biologic concept. Thus, O. F. Meyerhof was honoured with the Noble Prize for his discovery of the fixed relationship between the consumption of oxygen and the metabolism of lactic acid. Meyerhof presented data that muscle cells import lactic acid to restore glycogen storages, as well ‘burn’ lactic acid for ATP recovery. The ratio of imported lactic acid to burned lactic acid was determined to be between 6:1 and 4:1 [67]. The data opened an ancient biochemical dilemma: How can enzymes catalyse degradation and synthesis at the same time in the same cell? (Figure 4).

However, this dilemma was manmade by applying well-established chemical illustrations to a biological process. The “new” law of nature is key. The tentative Fourth Law of Thermodynamics states that a flow of energy is sufficient to form ordered structures [2,3,4]. The Glansdorff–Prigogine principle claims that the organism must compensate for the production of entropy by emitting entropy [2].

The physical quantity of Meyerhof´s imported lactic acid is (mol/s). Lactic acid flows (mol/s) into the Citric Acid Cycle 1.1 and is not present as a fixed concentration [mol/L]. The original Citric Acid Cycle covered the ‘entropy-emitting’ aspect of the Fourth Law of Thermodynamics. Imported lactic acid is “burned” to further emit carbonic acid and hydrogen (mol/s). The Citric Acid Cycle 1.1 adds the ‘entropy-producing’ aspect. Malic acid (mol) is produced within a dynamic context of emitting entropy. For the degradation of lactic acid and production of malic acid, the cycle acts in the same catalytic direction. Moreover, the Citric Acid Cycle 1.1 directly links the ‘burning’ of lactic acid and production of malic acid at the beginning of the cycle. The ‘burning’ of lactic acid recovers the activity of mitochondrial LDH-h. Thus, the burning is obligatory for lactic acid import and, thereby, malic acid synthesis. As such, a biological or dynamic process cannot be followed in context of the well-established chemical equilibration reaction.

The rationales of the Citric Acid Cycle 1.1 were introduced in context of environmental stressors. The same rationales also apply to homeostasis. The balance between the flow generated by ‘burning’ lactic acid and the flow generated by ‘burning’ hydrogen to water defines malic acid synthesis, thereby defining glycogen storages. Chronic diseases, as well as treatment with psychopharmaceuticals, are linked with metabolic syndromes [68]. The reformation of the basics in metabolism opens up mechanisms to metabolic syndromes. For example, major depression is associated with decreased glycogen storages, suggesting an imbalance of the flows in favour of ‘burning’ hydrogen, resulting in decreased malate synthesis [54]. Interestingly, treatment of depression with sodium/serotonin reuptake inhibitors (SSRI) further diminishes glycogen storages. One of the side effects of SSRI treatment is bleeding, indicating that the signalling pathway of thrombocyte glycogenolysis is pharmacologically knocked out by SSRI treatment [69].

The Citric Acid Cycle 1.1 only covers lactic acid as a substrate. The discovery of further Citric Acid Cycles and their dynamic interactions is necessary to functionally connect the massive changes in homeostasis triggered by olanzapine treatment of schizophrenia. Olanzapine treatment slowly changes into a glycogen-rich fast muscle fibre type. In addition, olanzapine also causes a switch to fatty acid oxidation and is associated with massive weight gain and reduced free fatty acid levels in blood during hyperglycaemia [70,71,72,73]. Our next manuscript will introduce the Citric Acid Cycle 2.1 as a cycle balancing fatty acid synthesis and beta-oxidation.

Unfortunately, the majority of scientific literature has failed to differentiate between carboxylates and carboxylic acids. Whether it is simply an inaccuracy in the nomenclature of basic organic molecules or a misunderstanding that carboxylate is not a synonym of carboxylic acid, both exclude differentiation between the pool of dicarboxylates used as substrates of ATP synthase and the flow of cycling carboxylic acids in the Citric Acid Cycle. ATP synthase, together with the pool of dicarboxylates, belong to the flow of energy driven by ETC, whereas the Citric Acid Cycle is driven by the metabolism of lacH. These two distinct flows of energy and material are far from being fully understood.

## Abbreviations

lacH	lactic acid
pyrH	pyruvic acid
ETC	electron transport chain
PTC	proton transport chain
SDH	succinate dehydrogenase
MDH	malate dehydrogenase
PDHc	pyruvate dehydrogenase complex
PC	pyruvate carboxylase
